# P-2019. Retrospective Cohort Study on use of Monoclonal Antibodies against COVID19 during Pregnancy

**DOI:** 10.1093/ofid/ofae631.2176

**Published:** 2025-01-29

**Authors:** Praveen Kumar Vikraman, Ahmed Shamayl, Shivanthidevi Gandhi, Rubab Sohail, Meredith Akerman, Cristina Sison, Henry Donaghy

**Affiliations:** Appalachian Regional Healthcare, Hazard, Kentucky; Northwell - Long Island Jewish Medical Center, New Hyde Park, New York; North Shore University Hospital, New Hyde Park, New York; Northwell Health, New Hyde Park, New York; Northwell Health, New Hyde Park, New York; Feinstein Institutes for Medical Research, Manhasset, New York; Northwell Health, New Hyde Park, New York

## Abstract

**Background:**

The safety and efficacy of monoclonal antibodies (MAB) against SARS-CoV-2 virus was studied in pregnant individuals with COVID19 infection. We examined adverse outcomes associated with MAB infusion in pregnant patients with COVID19 and compared mortality outcomes between the cohorts; pregnant patients with COVID19 who received MAB and pregnant patients with COVID19 who did not receive MAB.

**Methods:**

A cross-sectional retrospective chart review of pregnant patients with COVID19 treated in the Northwell Health System from January 2021 to January 2022 was conducted. Outcomes studied included comparing demographic and clinical characteristics amongst pregnant women who received MAB infusion vs. pregnant women who did not receive MAB infusion, the association between MAB infusion and adverse pregnancy outcomes within one month of MAB infusion, and the association between MAB infusion and 30-day all-cause mortality.

**Results:**

A total of 269 patients were studied. 49% were White, mean ± SD age was 31.8 (5.5) years, 26% were vaccinated for COVID19, 45% received MAB infusion, 3% reported adverse infusion related events, and 4% reported adverse pregnancy outcomes.

Subjects that received MAB infusion were more likely to be older (33 vs. 30.8 years, p=0.0008) and vaccinated (85% vs. 14%; p< 0.0001). Monoclonal antibody utilization was higher in Caucasian women compared to other racial and ethnic groups (p< 0.0001). In subjects that received MAB infusion, 9% (n = 11) reported an adverse pregnancy outcome. Only 1 subject passed away at 30 days (did not receive MAB infusion). All subjects receiving MAB survived at 30 days.

**Conclusion:**

The number of patients who received MAB and reported adverse infusion related events and adverse pregnancy outcomes within 1 month of MAB infusion were low. Patients who had been vaccinated and Caucasian patients were more likely to receive monoclonal antibody. Further study is needed to elucidate factors influencing the differential uptake of novel therapeutic options for the management of SARS-CoV-2, including access to care and factors influencing patient preferences in using new therapies.

**Disclosures:**

All Authors: No reported disclosures

